# Impact of acute sleep restriction on resting ventilation and cytokine levels in normobaric hypoxia, and their association

**DOI:** 10.1007/s00421-025-06105-5

**Published:** 2026-01-28

**Authors:** Louis David, Julianne Touron, Marie-Claire Erkel, Catherine Drogou, Anaïs Pontiggia, Vincent Beauchamps, Carine Malle, Olivier Nespoulous, Ulysse Comte, Alexandra Malgoyre, Fabien Sauvet, Beth A. Beidleman, Nathalie Koulmann, Danielle Gomez-Merino, Mounir Chennaoui, Pierre A. Fabries, HYPSOM Investigator Group, Haïk Ayounts, Catherine Bellec, Keyne Charlot, Paco Clavé, Françoise Gignoux-Huon, Mathias Guillard, Clémentine Jacques, Romaric Jérome, Emilie Louis-Delauriere, Théo Pinalie, Michael Quiquempoix, Aurélie Servonnet, Aurélie Trignol, Pascal Van Beers

**Affiliations:** 1 Académie de Santé des Armées (ACASAN), École du Val-de-Grâce (EVDG), Paris, France; 2 Hôpital Régional d’Instruction Des Armées Clermont-Tonnerre, Brest, France; 3 Institut de Recherche Biomédicale Des Armées (IRBA), Brétigny-Sur-Orge, France; 4 UMR 7330, Université Paris-Cité, Hôpital Hôtel-Dieu, Paris, France; 5 Hôpital National d’Instruction Des Armées Percy, Clamart, France; 6 Laboratoire de Biologie de L’Exercice pour la Performance et la Santé (LBEPS), Université Évry, IRBA, Université Paris-Saclay, Évry, France; 7 US Army Research Institute of Environmental Medicine (USARIEM), 10 General Greene Ave., Natick, MA 01760 USA

**Keywords:** Normobaric hypoxia, Sleep, Ventilation, Cytokines

## Abstract

The effect of sleep restriction (SR) on the physiological responses to normobaric hypoxia (NH) remains unknown. This study examined whether acute SR the night prior to a 5-h exposure to 3,500 m NH impacts (i) the resting ventilatory response, (ii) circulating pro- and anti-inflammatory cytokines, and (iii) their potential association. Seventeen healthy men (31 ± 7 yr; 77.1 ± 8.5 kg) were exposed to 5 h of NH (F_I_O_2_ = 13.6%, 3,500 m) in a randomized crossover design following one night of habitual (> 6 h) or restricted (≤ 3 h) sleep, separated by at least one week. Minute ventilation (V̇E), carbon dioxide output (V̇CO_2_), oxygen uptake (V̇O_2_), tidal volume (VT), and respiratory rate (RR) were assessed after 1.5 h of NH. Plasma cytokines (TNF-α, IL-8, IL-10) were measured following 1.5 h and 5 h of NH. SR increased VT (0.67 ± 0.34 *vs*. 0.63 ± 0.33 L, *p* = 0.031) following 1.5 h of NH. TNF-α was elevated following SR at both 1.5 h (0.98 ± 0.38 *vs.* 0.86 ± 0.41 pg/mL, *p* = 0.012) and 5 h (1.00 ± 0.45 *vs.* 0.79 ± 0.35 pg/mL, *p* = 0.004) of NH exposure. IL-10 levels increased after 5 h of NH following SR (0.94 ± 0.65 *vs.* 0.78 ± 0.73 pg/mL, *p* = 0.040). Ventilatory responses (VT, V̇E, V̇O_2_, V̇CO_2_, RER, V̇E/V̇O_2_, V̇E/V̇CO_2_, P_ET_O_2_, V̇A, SpO_2_) positively correlated with IL-10 after 1.5 h of NH (r ranged from 0.433 to 0.659, *p* < 0.05). P_ET_CO_2_ negatively correlated with IL-10 (r = −0.411, *p* = 0.033). Acute SR modulates the ventilatory and inflammatory responses to NH which may influence altitude tolerance. IL-10 may influence ventilatory responses to NH. In this context, IL-10 is associated with ventilatory responses to NH.

## Introduction

Due to the decrease in inspired oxygen pressure at high altitude (HA) (Hurtado 1964), there is an increase in ventilation triggered by the hypoxic ventilatory response (HVR) (Bärtsch and Gibbs 2007; Cogo 2011; Burtscher et al. 2022), an increase in heart rate (HR) due to sympathetic activation and vagal withdrawal (Bärtsch and Gibbs 2007; Burtscher et al. 2022), and an increase in hematocrit due to a reduction in plasma volume (Burtscher et al. 2022). All of these acute changes increase oxygen (O_2_) delivery to the working muscles and brain in an O_2_-limited environment. However, in real-life situations, exposure to hypoxia rarely occurs in isolation and is almost always accompanied by sleep restriction (SR), whether before exposure (such as in jet lag, night missions for the military, or search and rescue operations), or during mountain treks where nights are disturbed by harsh conditions in shelters or tents (Fabries et al. 2022).

Our previous review (Fabries et al. 2022) aimed to identify how sleep loss may impact ventilatory responses to hypoxia. Rault et al. (2020) demonstrated that one night of sleep deprivation at sea level (SL) reduces inspiratory endurance through altered cortical contribution to respiratory motor output (Rault et al. 2020). Another previous study reported that sleep restriction at SL could also have consequences on peripheral respiratory muscle fatigue (Phillips 1985), leading to a small but significant decrease in forced vital capacity and maximal voluntary ventilation after 27 h of wakefulness (Cooper and Phillips 1982). Exposure to normobaric hypoxia (NH) alone without SR has also been shown to increase respiratory muscle fatigue during three 15-min hyperpnoea tests (NH; pulse oximetry (SpO_2_) = 80%) (Verges et al. 2010). At the brain level, it was found that simulated acute exposure to moderate NH (3,000 m) without SR also alters the functional connectivity of cerebral networks (Liu et al. 2022). Taken together, the above data suggest that prior sleep loss could interfere with hypoxic ventilatory responses, through a worsening of central or peripheral fatigue. In addition, individuals that demonstrate a poor HVR have been reported to experience greater symptoms of acute mountain sickness (AMS) (Nespoulet et al. 2012; Richalet et al. 2012; Burtscher et al. 2019).

In our previous review, we also hypothesized that the link between the two constraints, hypoxia and sleep loss, might be inflammation (Fabries et al. 2022). Indeed, it is well-established that sleep deprivation/restriction is associated with subclinical changes in the levels of pro- and anti-inflammatory cytokines (Mullington et al. 2009; Chennaoui et al. 2011; Irwin et al. 2016; Sauvet et al. 2017). For example, after 48 h at an altitude of 3,800 m, blood levels of both pro- and anti-inflammatory cytokines (including Interleukin (IL)-1β, IL-6, IL-8, IL-10, and Tumor Necrosis Factor alpha (TNF-α)) have been shown to increase (Lundeberg et al. 2018) from SL values. Similar increases have been observed 24 h after passive ascent to 3,883 m, where blood levels of TNF-α, IL-1β, and IL-6 rose, sometimes accompanied by increased IL-1β mRNA expression (Kammerer et al. 2020). Cytokine responses have also been linked to individual differences in acute high-altitude illness syndromes (Ullah et al. 2025).

Interestingly, there is evidence that cytokines may be involved in the control of breathing (Fung 2015; Giannakopoulou et al. 2019). A recent study on mice demonstrated that IL-10 can increase the drive to breathe and can produce a slow, deep pattern of breathing without changing the inspiration to expiration ratio (Giannakopoulou et al. 2019). These authors suggested both central and peripheral effects of IL-10 action. All of the above data suggest that upon exposure to NH, SR the previous night may influence cytokine responses and ultimately alter altitude tolerance.

We hypothesized that SR prior to NH exposure may influence ventilatory responses as well as the blood concentrations of pro-and anti-inflammatory cytokines, and that there would be a relationship between the two variables. To validate these hypotheses, we conducted a study involving 17 healthy males to assess resting ventilation and pro- and anti-inflammatory blood cytokine concentrations during a 5-h exposure to NH at a simulated altitude of 3,500 m (inspired oxygen fraction (F_I_O_2_) = 13.6%) following a night of habitual sleep (total sleep time (TST) > 6 h) and a night of SR (TST ≤ 3 h). We further hypothesized that changes in ventilatory parameters may be related to changes in cytokine levels.

## Materials and methods

### Ethics

The protocol was approved by the "*Comité de protection des personnes* Ile-de-France VIII" on 3 August 2022 (IDRCB no.: 2022-A00464-39, internal no.: 2021PBMD09). All procedures were conducted in accordance with the Declaration of Helsinki and institutional protocols. All participants provided written informed consent before participation. Participants were included at the French Armed Forces Biomedical Research Institute—IRBA—Brétigny-sur-Orge (Fr-91220). Protocol has been registered in the clinical trial registry (NCT05563688, https://clinicaltrials.gov/study/NCT05563688).

### Participants

Seventeen healthy male participants were included. The mean age of the participants was 31 ± 7 years, height 1.80 ± 0.07 m, body weight 77.1 ± 8.5 kg and body mass index (BMI = body weight/height^2^) 23.8 ± 1.7 kg.m^−2^.

We included participants without a history of sleep disturbances, verified by the Pittsburg Sleep Quality Index < 5, with a usual time in bed (TIB) ≥ 6 h (Buysse et al. 1989). Exclusion criteria included being female (due to differences in ventilatory response to hypoxia depending on the phase of the menstrual cycle (Richalet et al. 2020)), having an active medical pathology or a significant clinical abnormality at the inclusion visit (these two latter items were also reviewed and confirmed by the study physician at the onset of every follow-up visit). Exposure to an altitude of 3,500 m or more in the previous three months was also an exclusion criterion, due to the risk of pre-acclimatization (Schneider et al. 2002). All lowland participants additionally confirmed having had no recent exposure to moderate altitudes in the 12 weeks preceding the study.

### Experimental design

Participants were exposed to two experimental conditions in a NH tent using a cross-over design, with a wash-out period of at least 7 days:

- NH (F_I_O_2_ = 13.6%, simulated 3,500 m altitude) after a habitual night’s sleep (HS condition, TST > 6 h, in bed from 10 pm to 6 am);
- NH (F_I_O_2_ = 13.6%, simulated a 3,500 m of altitude exposure) after a night of sleep restriction (SR condition, TST ≤ 3 h, in bed from 3 to 6 am).

After the inclusion visit, participants were randomly assigned to one of the two groups (A or B) by drawing sealed envelopes. The groups differed only in the order in which the conditions were administered (Group A: HS followed by SR, *vs.* Group B: SR followed by HS).

**Fig. 1:**
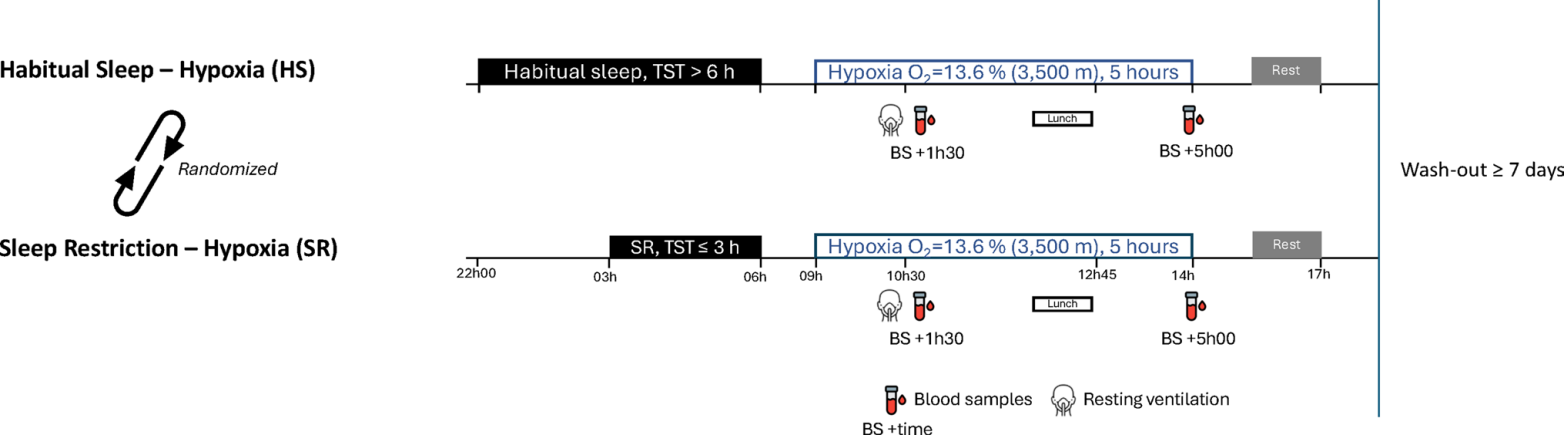
Study design. Participants were exposed to 5 h of normobaric hypoxia (NH) (F_I_O_2_ = 13.6%, 3,500 m) from 9 am to 2 pm, following either a habitual night of sleep (HS, total sleep time (TST) > 6 h) or a night of sleep restriction (SR, TST ≤ 3 h) at a sleep laboratory at SL. The conditions were randomized, with a washout period of at least 7 d between them. Resting ventilation was measured after 1.5 h of NH exposure, and blood samples were collected at 1.5 h and 5 h of exposure to NH

Participants were in the tent from 9 am to 2 pm, and were always at rest (i.e. non exercising) (Fig. 1). The various measurements are described below.

This study is part of a larger protocol, HYPSOM. The primary objective of this protocol was to assess the effects of SR on cognitive responses to NH at an altitude of 3,500 m (Fabries et al. 2024). The main outcome measure was reaction time on the Psychomotor Vigilance Task (PVT), assessed after 30 min of exposure to NH. The sample size calculation for this parameter indicated that at least 14 participants were required to detect a difference greater than 30 ms, assuming a standard deviation of 22 ms, with a significance level (alpha) of 5% and a statistical power of 90%.

### Normobaric hypoxia exposure

The experiment was conducted in a 40 m^3^ normobaric hypoxic tent (Sporting Edge, Basingstoke, UK) located in Brétigny-sur-Orge (at 75 m above SL). Four participants were placed in the tent during each session. The settings were identical for both conditions. The tent was designed to ensure constant lighting (200 lx), stable temperature (21 °C), and low ambient noise levels. The F_I_O_2_ was set at 13.6%, corresponding to an equivalent altitude of approximately 3,500 m (11,500 feet). The system maintained the oxygen concentration within a range of ± 0.1% (or ± 40 m in altitude) from a single injection point, with continuous air mixing inside the tent via a dedicated fan. The inspired carbon dioxide fraction (F_I_CO_2_) was kept < 0.08% (800 parts per million) through a controlled air exchange rate, with uniform ventilation in the tent. The concentrations of both gases (F_I_O_2_ and F_I_CO_2_) were continuously monitored using a calibrated O₂ gas analyzer GA200 (iWorks, New Hampshire, USA).

### Sleep conditions

Prior to their admission to the sleep laboratory and for 1 week (7 days), the participants wore an actigraph (MotionWatch 8, CamNTech, Papworth Everard, UK) and were instructed to maintain regular sleep–wake behavior with their usual 7 to 8 h of sleep (i.e., in bed around 10 pm until 6 am) and avoid late hours. They then wore the actigraph throughout the study and adhered to the same instructions during the washout period. TST was therefore measured using the MotionWatch 8 (CamNTech), an actigraphy device validated against gold-standard polysomnography (Elbaz et al. 2012). The actigraph data enabled us to verify that the participants were complying with the instructions. Participants arrived at the sleep laboratory the previous day (at 7–8 pm). In HS condition, the TST was > 6 h (in bed from 10 pm to 6 am); in SR condition the TST was ≤ 3 h (in bed from 3 to 6 am). During sleep restriction, participants were kept awake through non-physical, distraction-based activities (i.e., board games as well as time allocated for meals, personal hygiene, bedroom preparation, and hypoxic tent setup), following an identical schedule to ensure they remained awake in a comparable manner. Nights were spent in the same laboratory apartment at SL, and a researcher was present at all times to monitor participants’ wakefulness, particularly during SR.

### Resting ventilation

Resting ventilation was assessed once, 1.5 h after the start of NH exposure (i.e., at 10:30 am). Participants were seated in a comfortable chair while breathing through a well-fitted mask (7450, Hans Rudolph, Inc., Shawnee, KS, USA) for 10 min. Respiratory rate (RR, breaths per minute), tidal volume (VT, L), carbon dioxide output (V̇CO_2_, mL/min), oxygen uptake (V̇O_2_, mL/min), end-tidal O_2_ pressure (P_ET_O_2_, mmHg), end-tidal CO_2_ pressure (P_ET_CO_2_, mmHg), heart rate (HR, bpm), and arterial oxygen saturation from pulse oximetry (SpO_2_, %) were measured using a Quark CPET (Cosmed®, Rome, Italy) daily calibrated, and an ear clip pulse oximetry sensor (model 8000Q2, Nonin®, Plymouth, MN) placed at the earlobe. The mean values of the last 5 min of recording were used for statistical analysis. The device also calculated the minute ventilation (V̇E = RR x VT, L/min), the respiratory exchange ratio (RER = V̇CO_2_/V̇O_2_), and ventilatory equivalents for oxygen and carbon dioxide (V̇E/V̇O_2_ and V̇E/V̇CO_2_). We calculated alveolar ventilation (V̇A, L/min) using the following formula (West and Luks 2021): V̇A = V̇E × (1 − VD/VT), with VD/VT = (P_ET_CO_2_ − PeCO_2_) / P_ET_CO_2_. PeCO_2_ was calculated by the Quark CPET from expired-gas measurements collected.

### Blood samples

During each visit under HS and SR conditions, blood samples were collected via venipuncture from the antecubital vein after 1.5 h of NH exposure (BS + 1h30, at 10:30 am, immediately following the ventilatory measurements) and again after 5 h of NH exposure (BS + 5h00, at 2 pm, immediately prior to exiting the hypoxic tent).

### Hemoglobin and hematocrit

An automated system (XN-L, Sysmex France®, Villepinte, France) was used to determine the plasma hemoglobin concentration ([Hb]) (g/dL) and hematocrit (Hct) level (%) at BS + 1h30 and BS + 5h00. We then calculated the change in plasma volume (PV) between BS + 1h30 and BS + 5h00 using the Dill and Costill equation (Dill and Costill 1974).

### Cytokines

At each time point (BS + 1h30 and BS + 5h00), one 4 mL sample was collected from one EDTA tube. After centrifugation, plasma samples were aliquoted and stored at -80 °C until analysis. Plasma IL-8, IL-10, TNF-α, IL-1β and IL-6 levels (Simoa® CorPlex™ Cytokine Panel-1 5-Plex Kit, ref.85–0353, Quanterix, USA) were analyzed using a SP-X Imaging and Analysis System™ (Quanterix, USA), following the manufacturer’s instructions. Each sample was analyzed in duplicate. Laboratory personnel conducting cytokine measurements were blinded to group allocation. The TNF-α/IL-10 ratio was calculated at each measurement point. The functional lower limits of quantification (LLOQ) for IL-8, IL-10 and TNF-α were 1.56 pg/mL, 0.10 pg/mL and 0.39 pg/mL, respectively. Average intra-assay coefficients of variation for IL-8, IL-10 and TNF-α were 13.9%, 10.0% and 10.1%, respectively. Average inter-assay coefficients of variation for IL-8, IL-10 and TNF-α were 9.0%, 5.1% and 6.9%, respectively.

After performing the assays, we were unable to analyze the results for the concentrations of IL-1β and IL-6 as the values were below the functional LLOQ (0.10 pg/mL and 0.59 pg/mL, respectively).

### Diet

During this study, participants were allowed to drink ad libitum. They were only allowed to eat the same standardized breakfast and lunch in both experimental conditions, and this food intake was the subject of a recent published study (Clavé et al. 2025). Habitual coffee consumers were allowed to drink one coffee only at breakfast, and in an identical manner under both the HS and SR conditions. Alcohol consumption was not permitted. Moreover, participants were not permitted to engage in any physical exercise on the day preceding each experimental visit.

### Statistics

All variables were checked for normality using the Shapiro–Wilk test. The paired t-test or the Wilcoxon signed-rank test was used for paired samples, depending on the normality of the data. Initially, we analyzed ventilation parameters between the SR and HS conditions. Then, we analyzed blood parameters ([Hb], Hct, and cytokine concentrations) between the SR and HS conditions (BS + 1h30 *vs.* BS + 1h30, and BS + 5h00 *vs.* BS + 5h00) and within the SR and HS conditions (BS + 5h00 *vs.* BS + 1h30). Pearson’s correlation analyses were performed only between respiratory variables and cytokine concentrations obtained at the same time point (BS + 1h30), with data from HS and SR pooled. All data are expressed as mean ± standard deviation (SD). *p*-value < 0.05 was considered to be significant. Statistical analyses were performed using the free software JAMOVI (version 2.5.2.0) and Rj Editor module.

The primary ventilatory endpoint was VT, compared between the two experimental conditions (HS and SR). The primary blood endpoint was the plasma IL-10 concentration, compared between HS and SR at BS + 5h00, the time point assumed to be optimal for detecting the hypoxia-related effect. Based on our a priori hypothesis that ventilatory responses represent the main physiological outcome of interest—particularly VT at this simulated altitude (Cogo 2011)—and that IL-10 may be associated with these ventilatory changes (Giannakopoulou et al. 2019), we prespecified a hierarchical relationship between the two primary endpoints. To control the family-wise type I error rate, a hierarchical testing procedure was applied: VT was tested first at a two-sided α level of 0.05, and the IL-10 endpoint was subsequently tested only if the VT test reached significance. No additional multiplicity correction was required within this framework. All other ventilatory variables and additional blood markers were considered secondary endpoints. For these outcomes, *p*-values were adjusted for multiple comparisons using the Holm procedure (Holm 1979), applied separately within each family of tests (ventilatory parameters, physiological parameters, cytokine concentrations, hematological variables). Correlation analyses between ventilatory parameters and IL-10 levels were also considered secondary analyses, and their *p*-values were similarly corrected using the Holm method (Holm 1979).

The figure was generated using Microsoft® Excel (version 16.96.1).

## Results

### Effectiveness of sleep restriction

The mean TST during HS were 6.5 ± 0.4 h, and 2.4 ± 0.2 h during SR. Mean sleep efficiency index (SEI = TST/TIB), an index of sleep quality, was equal to 84.2 ± 32.1%, with no difference between conditions.

### Effectiveness of hypoxia exposure

The SpO_2_ values in the HS and SR conditions were 89.83 ± 3.34% and 89.83 ± 3.72%, respectively, with no significant difference between conditions (*p*_Holm_ = 0.995). HR was lower in the HS condition than in the SR condition (61.81 ± 7.76 *vs.* 63.91 ± 7.10 bpm, *p*_Holm_ = 0.042).

### Resting ventilation

After 1.5 h of NH exposure, VT was lower in the HS condition compared to the SR condition (*p* = 0.031) (Table 1). After correction of the *p*-values, no significant differences were observed for the other ventilatory parameters.

**Table 1:** Resting ventilation parameters under normobaric hypoxia (NH) (F_I_O_2_ = 13.6%, 3,500 m) after habitual sleep (HS, total sleep time (TST) > 6 h) and after sleep restriction (SR, TST ≤ 3 h) (n = 17)

Parameters	HS	SR	*p* (raw)	*p* (Holm)
VT (L)	0.63 ± 0.33 [0.46–0.80]	0.67 ± 0.34* [0.50–0.85]	0.031	–
RR (bpm)	12.98 ± 3.09 [11.40–14.60]	13.33 ± 3.26 [11.70–15.00]	0.426	0.852
V̇E (L/min)	7.61 ± 2.76 [6.19–9.03]	8.34 ± 3.21 [6.69–9.99]	0.070	0.560
V̇O_2_ (mL/min)	241.08 ± 56.62 [212.00–270.00]	259.79 ± 56.93 [231.00–289.00]	0.093	0.651
V̇CO_2_ (mL/min)	191.28 ± 59.97 [161.00–222.00]	200.11 ± 54.29 [172.00–228.00]	0.284	0.852
RER	0.79 ± 0.08 [0.74–0.83]	0.77 ± 0.06 [0.74–0.80]	0.033	0.297
V̇E/V̇O_2_	27.05 ± 5.21 [24.40–29.70]	27.97 ± 6.14 [24.80–31.31]	0.138	0.651
V̇E/V̇CO_2_	34.29 ± 3.98 [32.20–36.30]	36.30 ± 5.27 [33.60–39.00]	0.024	0.240
P_ET_O_2_ (mmHg)	58.70 ± 5.13 [56.10–61.30]	58.53 ± 5.22 [55.80–61.20]	0.862	0.862
P_ET_CO_2_ (mmHg)	33.38 ± 3.79 [32.20–35.60]	33.10 ± 3.43 [31.30–34.90]	0.120	0.651
V̇A (L/min)	5.44 ± 2.45 [4.18–6.70]	5.97 ± 2.63 [4.62–7.32]	0.094	0.651

*bpm* breaths per minute, *P*_*ET*_*O*_*2*_ end-tidal O_2_ pressure, *P*_*ET*_*CO*_*2*_ end-tidal CO_2_ pressure, *RER* respiratory exchange ratio, *RR* respiratory rate, *SpO*_*2*_ oxygen saturation from pulse oximetry, *V̇A* alveolar ventilation, *V̇E* minute ventilation, *V̇E/V̇CO*_*2*_ ventilatory equivalent for carbon dioxide, *V̇E/V̇O*_*2*_ ventilatory equivalent for oxygen, *V̇O*_*2*_ oxygen uptake, *V̇CO*_*2*_ carbon dioxide output, *VT* tidal volume. Mean ± SD, and [95% confidence interval]

*p_raw_ < 0.05 is difference between conditions (HS *vs.* SR)

### Blood samples

#### Hemoglobin and hematocrit

There were no differences in [Hb] levels after 1.5 h (BS + 1h30) and 5 h (BS + 5h00) of NH exposure between HS and SR conditions (15.6 ± 0.9 *vs.* 15.4 ± 1.1 g/L, and 15.6 ± 0.9 *vs.* 15.6 ± 0.9 g/L, respectively, *p*_Holm_ > 0.05). There were also no differences in Hct after 1.5 h (BS + 1h30) and 5 h (BS + 5h00) of NH exposure between SR and HS conditions (46.4 ± 2.1 *vs.* 46.0 ± 2.9%, and 46.2 ± 2.9 *vs.* 46.2 ± 2.1%, respectively, *p*_Holm_ > 0.05). Additionally, there were no differences in the change in plasma volume (ΔPV) between BS + 1h30 and BS + 5h00 for HS (-0.009 ± 0.019) and SR conditions (-0.004 ± 0.019) (*p*_Holm_ > 0.05).

#### Cytokines

##### TNF-α

**Fig. 2:**
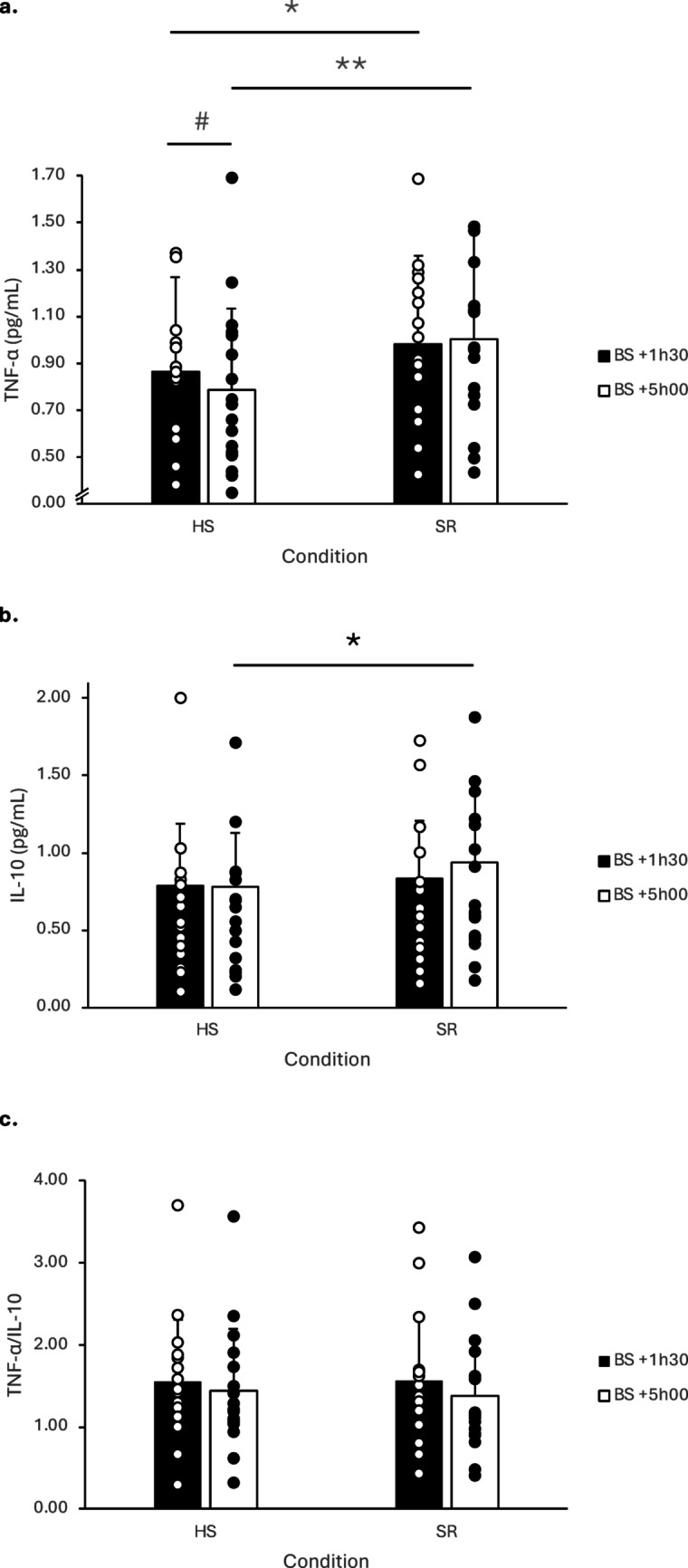
Plasma concentrations of TNF-α (**a**), IL-10 (**b**), and TNF-α/IL-10 ratio (**c**), after 1h30 (BS + 1h30, black column) and 5h00 (BS + 5h00, white column) of normobaric hypoxia (NH) exposure (F_I_O_2_ = 13.6%, 3,500 m) after one night of habitual sleep (HS, total sleep time (TST) > 6 h) and after one night of sleep restriction (SR, TST ≤ 3 h) (n = 17). TNF-α, tumor necrosis factor alpha; IL-10, interleukin 10. Mean ± SD and individual values. *p_raw_ < 0.05, *p*_Holm_ < 0.05, *p*_Holm_ < 0.01, are difference between HS *vs.* SR; *p*_Holm_ < 0.05 is difference between BS + 1h30 *vs.* BS + 5h00

In the HS compared to the SR condition, the TNF-α concentration was lower at BS + 1h30 (*p*_Holm_ = 0.012) and BS + 5h00 (*p*_Holm_ = 0.004) (Fig. 2a). In the HS condition, the concentration was higher at BS + 1h30 compared to BS + 5h00 (*p*_Holm_ = 0.012), but there were no difference in TNF- α between time points in the SR condition (*p*_Holm_ = 0.556).

##### IL-10

The IL-10 concentration was lower at BS + 5h00 in HS *vs.* SR condition (*p* = 0.040) (Fig. 2b). No differences were found at BS + 1h30 between the two conditions (*p*_Holm_ = 1), nor were any differences observed within the HS or SR conditions (*p*_Holm_ = 1 and 0.078, respectively).

##### TNF-α/IL-10 ratio

No differences were observed between the HS and SR conditions, nor between the measurement time points within either condition (*p*_Holm_ > 0.05; Fig. 2c).

##### IL-8

There were no differences in IL-8 between HS *vs.* SR conditions at either time point (5.14 ± 2.52 *vs.* 5.52 ± 2.78 pg/mL, *p*_Holm_ = 0.663 for BS + 1h30 and 5.50 ± 2.14 *vs.* 5.81 ± 2.56 pg/mL, *p*_Holm_ = 0.663 for BS + 5h00), In addition IL-8 did not change between time points in the HS (*p*_Holm_ = 0.392) and SR conditions (*p*_Holm_ = 0.663).

### Correlations analysis

When the two conditions were pooled (HS and SR), at BS + 1h30, positive correlations were observed between plasma IL-10 concentrations and resting ventilation parameters, particularly VT, V̇E, V̇O_2_, V̇CO_2_ RER, V̇E/V̇O_2_, V̇E/V̇CO_2_, P_ET_O_2_, V̇A and SpO_2_ (Table 2). P_ET_CO_2_ was negatively correlated with plasma IL-10 concentration.

There were﻿ ﻿no significant correlation between resting ventilatory parameters and TNF-α and IL-8 concentrations (data not shown).

**Table 2:** Pearson correlation analysis between resting ventilation parameters and IL-10 plasma concentrations under normobaric hypoxia (NH) (F_I_O_2_ = 13.6%, 3,500 m) after 1.5 h (BS + 1h30) of NH exposure, pooling the two sleep conditions (habitual sleep HS, total sleep time (TST) > 6 h, and sleep restriction SR, TST ≤ 3 h) (Pearson correlation coefficient with the [95% confidence interval]) (n = 34)

Ventilatory parameters (HS + SR pooled)	IL-10 concentration at BS + 1h30 (HS + SR pooled)			
	Pearson correlation coefficient, r	95% Confidence interval	*p* (raw)	*p* (Holm)
VT	0.650*	[0.400–0.810]	< 0.001	0.011
V̇E	0.633*	[0.375–0.800]	< 0.001	0.011
V̇O_2_	0.433*	[0.111–0.673]	0.011	0.025
V̇CO_2_	0.579*	[0.300–0.767]	< 0.001	0.011
RER	0.516*	[0.216–0.727]	0.002	0.012
V̇E/V̇O_2_	0.613*	[0.347–0.788]	< 0.001	0.011
V̇E/V̇CO_2_	0.460*	[0.144–0.691]	0.006	0.025
P_ET_O_2_	0.474*	[0.162–0.700]	0.005	0.025
P_ET_CO_2_	−0.411*	[−0.658– −0.085]	0.016	0.025
V̇A	0.659*	[0.401–0.811]	< 0.001	0.011
SpO_2_	0.453*	[0.135–0.686]	0.007	0.025

*HS* habitual sleep, *IL-10* interleukin 10, *P*_*ET*_*O*_*2*_ end-tidal O_2_ pressure, *P*_*ET*_*CO*_*2*_ end-tidal CO_2_ pressure, *RER* respiratory exchange ratio, *SpO*_*2*_ oxygen saturation from pulse oximetry, *SR* sleep restriction, *V̇A* alveolar ventilation, *V̇E* minute ventilation, *V̇E/V̇CO*_*2*_ ventilatory equivalent for carbon dioxide, *V̇E/V̇O*_*2*_ ventilatory equivalent for oxygen, *VT* tidal volume

*p_Holm_ < 0.05

## Discussion

To our knowledge, this is the first study isolating the effects of acute SR on ventilatory and blood cytokine responses to acute NH exposure (F_I_O_2_ = 13.6%, 3,500 m), and their association in this context.

The main finding is that a single night of SR is sufficient to impact the ventilatory response to NH. Specifically, we observed an increase in VT. Interestingly, this result was opposite of our hypothesized decrease in ventilatory parameters following SR. Regarding cytokine levels, the second key result indicates that SR induces changes in circulating concentrations of both pro- and anti-inflammatory cytokines, which are themselves exacerbated by hypoxia (Lundeberg et al. 2018; Kammerer et al. 2020). More precisely, following one night of SR, the pro-inflammatory cytokine, TNF-α, was higher compared to HS during a 5-h exposure to NH. Conversely, although no difference was observed in levels of the anti-inflammatory cytokine, IL-10, after 1.5 h of NH between the two sleep conditions, higher IL-10 concentrations were detected after 5 h of NH exposure in SR participants. Interestingly, under these hypoxic conditions, all ventilatory parameters were correlated with IL-10 concentrations-a cytokine recently identified as a regulator of part of the hypoxic ventilatory response in animal models (Giannakopoulou et al. 2019)—regardless of sleep conditions.

We measured resting ventilation only once during this experiment. This measurement was taken 1.5 h after the start of the NH exposure. The timing was chosen to avoid the acute rise in ventilation initiated by hypoxic exposure followed by a secondary roll-off in ventilation termed the hyopxic ventilatory depression, which typically occurs in the first 30 min of exposure (Teppema and Berendsen 2014). After this initial phase of changes in ventilation during an acute hypoxic exosure, a gradual increase in ventilation occurs over several days, which is characteristic of ventilatory acclimatization (Ainslie et al. 2013). Under hypoxic conditions, there is an increase in V̇E (Laciga and Koller 1976; Dempsey and Forster 1982; Burtscher et al. 2019), which, at this simulated altitude of 3,500 m, is primarily driven by the well-known increase in VT (Cogo 2011). Above this altitude, RR increases significantly (Cogo 2011). Interestingly, in this study, which combined hypoxic exposure and sleep restriction, VT continues to increase without any significant change in V̇E. However, even in the absence of an increase in V̇A, our results demonstrate that when NH is combined with SR, hyperventilation and increased HR [the latter confirms our previous results at different measurement points (Fabries et al. 2024)] are two compensatory mechanisms implemented to ensure SpO_2_ levels similar to those observed after a normal night of 8 h in bed. In other words, acute SR combined with acute NH affects ventilation and HR in order to maintain SpO_2_ levels comparable to those observed under HS condition. However, because we do not have a control condition involving acute SR under normoxic conditions alone, it is not possible to determine which of the two factors—NH or SR—plays the predominant role.

We did not show, as we hypothesized, a decrease in ventilation under both constraints, whether of central or peripheral origin. This may be attributable to the SR model employed in our study, in which participants were subjected to a mild acute SR of 3 h, compared to the total sleep deprivation studies of Rault et al. (Rault et al. 2020) and Cooper and Phillips (Cooper and Phillips 1982). The normobaric hypoxic model used may also contribute to this difference. In our protocol, resting ventilation was measured at a simulated altitude of 3,500 m, where participants exhibited SpO_2_ values of ~ 90%. Verges et al. (Verges et al. 2010) demonstrated the effects of hypoxia on respiratory muscle fatigue using a more demanding model, combining lower SpO_2_ values (~ 80%) with voluntary hyperpnea protocol performed at 85% of maximal voluntary ventilation. In other terms, while SR modifies the ventilatory response to hypoxia, compensatory mechanisms remain effective in ensuring adequate ventilation at this simulated altitude of 3,500 m in resting participants.

Interestingly, in hypoxic conditions, when the HS and SR conditions were pooled, that is, regardless of the preceding sleep condition, ventilatory parameters were significantly and positively correlated with higher concentrations of the anti-inflammatory cytokine IL-10. Higher IL-10 levels were associated with higher ventilation (VT, V̇E, V̇O_2_, V̇CO_2_, RER, V̇E/V̇O_2_, V̇E/V̇CO_2_, P_ET_O_2_, V̇A) at BS + 1h30. We suggest that the relationship between these parameters may be mediated by the hypoxic peripheral chemoreflex response, characterized by increased ventilation (Prabhakar 2013) and activation of the sympathetic nervous system (Marshall 1994), thereby increasing arterial oxygen saturation, and consequently SpO_2_ (which in our study was positively correlated with IL-10 concentration). The sympathetic–immune interface would be mediated by the production of catecholamines (Elenkov et al. 1996). Sympathetic tone has also been shown to contribute to the crosstalk between sleep restriction and the immune system, including the production of the anti-inflammatory cytokine IL-10 (Lange et al. 2010). With regard to cytokine responses to hypoxia, elevated levels of TNF-α and IL-6 have been repeatedly observed after progressive or passive ascents to altitude—for example, by trekking or driving—up to approximately 3,900 m above sea level. In some cases, increased levels of IL-10 have also been reported (Boos et al. 2016; Lundeberg et al. 2018). In our study, however, such a comparison—specifically, relative to a normoxic condition—is not possible. This is because we did not measure circulating cytokine concentrations under normoxia, either with or without SR. However, we did find time-dependent effects of hypoxia exposure (5 *vs.* 1.5 h) on TNF-α, with a decrease only in the HS condition. This is probably due to its circadian pattern and not due to time spent in hypoxia. High levels of TNF-α typically occur on awakening and during daylight hours and are followed by a decline throughout the day (Reinhardt et al. 2019; Jasim et al. 2024). When we consider effects of SR, higher concentrations of TNF-α are observed compared to HS following both 1.5 h and 5 h of NH exposure. Since we and others have not observed an increase in circulating TNF-α concentration after a night of nearly identical sleep restriction but at SL (Lekander et al. 2013; Sauvet et al. 2015), we suggest that the additional three and a half hours spent in NH may have exacerbated susceptibility to an inflammatory response during SR. The observed increase in TNF-α is most likely due to the concomittant (5 h *vs.* 1.5 h) increase of two stress biomarkers (Wright et al. 2015), alpha-amylase, which signals activation of the sympathetic response, and cortisol, which signals activation of the hypothalamic-pituitary-adrenocortical axis (Fabries et al. 2024). We have previously demonstrated this increase in both stress biomarkers in saliva by studying their changes from the time of entry into the hypoxic chamber until exit (Fabries et al. 2024). On the other hand, the significant effect of SR on plasma IL-10 observed after 5 h of NH and not after 1.5 h, is likely a consequence of the significant effect of SR on the inflammatory response. During SR, this likely prevented the increase in the pro-inflammatory cytokine TNF-α, which showed no variation between the two measurement time points in the SR condition.

Several studies have demonstrated that a reduced hypoxic ventilatory response as well as increased TNF-α and reduced IL-10 levels are associated with AMS (Liu et al. 2017; Wang et al. 2018; Burtscher et al. 2019; Guo et al. 2024). In the SR condition, participants were able to increase VT and HR to maintain SpO_2_ while also increasing the anti-inflammatory cytokine IL-10. A recent study in mice demonstrated that under hypoxia, part of the breathing response is controlled by IL-10, which is associated with deeper and slower breathing (Giannakopoulou et al. 2019). These authors evidenced lower V̇E and VT in knockout IL-10^−/−^ mice compared with wild-type IL-10^+/+^ mice exposed to a hypoxic normocapnia 3% CO_2_-10% O_2_ gas challenge, meaning that the IL-10 presence is associated with higher V̇E and VT. The authors attributed these effects of IL-10 to central and peripheral mechanisms. Our study demonstrates significant positive correlations between IL-10 concentrations and ventilatory parameters and SpO_2_ in healthy humans exposed to NH whether or not they are sleep restricted. We therefore suggest that, under normobaric hypoxia, high concentrations of the anti-inflammatory cytokine IL-10, positively correlated with improved ventilatory responses and SpO_2_ levels, and this increase could be associated with a reduced risk of AMS (Liu et al. 2017).

Finally, there were no significant differences in hemoglobin or hematocrit values, which is consistent with less than one day of exposure at this simulated altitude (Schlittler et al. 2021).

## Limits and perspectives

These results are specific to acute exposure to NH at simulated 3,500 m altitude, and not to hypobaric hypoxia. They remains potentially transferable (i.e., the ventilatory response) to real-world conditions, as it has recently been demonstrated that the differences in resting cardioventilatory parameters between acute short-term exposure (approximately 25 min) to hypobaric and normobaric hypoxia at an equivalent altitude of approximately 4,000 m are minima (Vinetti et al. 2025). Furthermore, one possible limitation could be the absence of a control group that would allow us to isolate the effects of hypoxia and sleep restriction, but the main objective of our study was to explore the combined effects of these two constraints. Another critical limitation could be the absence of a pre-exposure (T0) normoxic baseline for plasma cytokines concentrations under each sleep condition, but we deliberately chose not to take blood samples before entering the hypoxic chamber for logistical reasons. It will also be interesting in future studies to evaluate sleep restriction effects over longer duration in both simulated and real-world altitude conditions, to better assess its impact on AMS. In this study, the duration of exposure to NH was too short to elicit significant AMS. Indeed, recent findings indicate that the mean Lake Louise AMS score remains below the threshold (≥ 3, of which at least 1 point for headache) during a 5-h passive exposure to NH (F_I_O_2_ = 13.6%, 3,500 m) with or without a 3-h SR (Clavé et al. 2025). Future work should include larger cohorts, both male and female, which will also increase the statistical power of the study. Complementary approaches, such as studies on respiratory endurance (Rault et al. 2020), and brain imaging to assess blood–brain integrity, due to the potential role of IL-10 in modulating its permeability (Garcia et al. 2017; Lin et al. 2018; Barabási et al. 2023), may provide valuable insights into the underlying mechanisms.

## Conclusion

This study is the first to isolate the effects of SR on ventilatory and blood cytokine responses to acute NH in healthy men. It provides a foundation for understanding the interaction between sleep and acute responses to hypoxia, as well as the potential role of sleep in altitude illnesses. It also strengthens the evidence supporting the control of ventilation by IL-10 in humans under normobaric hypoxia.

Like the initial results of this protocol (Fabries et al. 2024), this study underscores the importance of considering sleep when analyzing and comparing results from hypoxia studies, particularly in terms of physiological, cognitive, and biological parameters. It would therefore be useful if future studies systematically indicated the amount of sleep, which can now be easily assessed by wearable devices.

## Data Availability

The data that support the findings of this study are available from the corresponding author Dr Pierre Fabries upon reasonable request and with permission of SSA.

## Abbreviations

AMS: Acute mountain sickness
BMI: Body mass index
BS: Blood sample
F_I_O_2_: Inspired oxygen fraction
HA: High altitude
Hb: Hemoglobin
Hct: Hematocrit
HR: Heart rate
HS: Habitual sleep
HVR: Hypoxic ventilatory response
IL-10: Interleukin-10
IL-8: Interleukin-8
LLOQ: Lower limit of quantification
NH: Normobaric hypoxia
PeCO_2_: Expired carbon dioxide pressure
P_ET_CO_2_: End-tidal CO_2_ pressure
P_ET_O_2_: End-tidal O_2_ pressure
PVT: Psychomotor vigilance task
RER: Respiratory exchange ratio
RR: Respiratory rate
SD: Standard deviation
SL: Sea level
SpO_2_: Oxygen saturation from pulse oximetry
SR: Sleep restriction
TIB: Time in bed
TNF-α: Tumor necrosis factor alpha
TST: Total sleep time
V̇A: Alveolar ventilation
V̇CO_2_: Carbon dioxide output
VD: Dead space
V̇E: Minute ventilation
V̇E/V̇CO_2_: Ventilatory equivalent for carbon dioxide
V̇E/V̇O_2_: Ventilatory equivalent for oxygen
V̇O_2_: Oxygen uptake
VT: Tidal volume
